# The diagnostic value of inflammatory markers in bone metastasis of prostate cancer at initial prostate biopsy

**DOI:** 10.3389/fonc.2025.1626358

**Published:** 2025-10-09

**Authors:** Xinyang Chen, Yu Li, Zhiqin Chen, Gansheng Xie, Huming Yin, Gang Li

**Affiliations:** Department of Urology, The First Affiliated Hospital of Soochow University, Suzhou, China

**Keywords:** high-sensitivity C-reactive protein/albumin ratio, prostate cancer, prostate biopsy, Gleason score, bone metastasis, fibrinogen

## Abstract

**Introduction:**

Accurate prediction of bone metastasis at diagnosis is crucial for optimizing management in prostate cancer (PCa) patients. While clinical parameters like PSA and Gleason score are established predictors, their accuracy is suboptimal. Systemic inflammation, reflected in biomarkers like the high-sensitivity C-reactive protein-to-albumin ratio (HAR), fibrinogen (FIB), and hemoglobin (HB), has emerged as a key player in cancer progression, yet its integration into clinical predictive tools remains underexplored.

**Methods:**

In this retrospective study of 803 newly diagnosed PCa patients, we developed and validated two nomograms for predicting bone metastasis. A baseline clinical model was constructed using total prostate-specific antigen (TPSA) and biopsy Gleason grade groups. An enhanced comprehensive model integrated these clinical parameters with inflammatory markers (HAR, FIB, HB). Model performance was rigorously assessed through discrimination (ROC analysis, AUC), calibration (calibration curves, Hosmer-Lemeshow test), and clinical utility (Decision Curve Analysis). Internal validation was performed via bootstrapping.

**Results:**

Multivariate analysis confirmed TPSA, FIB, HB, HAR, and Gleason grade groups as independent predictors of bone metastasis. The comprehensive model demonstrated significantly superior discriminative ability, achieving an AUC of 0.874 (95% CI: 0.845–0.902) compared to 0.830 (95% CI: 0.798–0.863) for the clinical model (Delong’s test, P < 0.01). This translated to a net improvement in reclassification (NRI: 8.96%) and overall predictive performance (IDI: 10.3%). The model was well-calibrated and provided a positive net benefit across a wide range of clinical threshold probabilities.

**Conclusion:**

We present a novel, internally validated nomogram that synergistically combines inflammatory and clinical markers to accurately predict bone metastasis in PCa at initial diagnosis. This practical and cost-effective tool has the potential to aid clinicians in risk stratification, guide personalized diagnostic imaging decisions, and ultimately help reduce unnecessary bone scans, particularly in resource-conscious settings. Our findings underscore the pivotal role of the systemic inflammatory response in PCa metastasis.

## 1 Introduction

Prostate cancer (PCa) is the most common malignant tumor of the urogenital system in men. It is the second most common malignant tumor in men worldwide, after lung cancer (1). Despite the fact that China’s incidence of PCa is still lower than that of Western nations, the disease’s incidence and mortality are rising annually as a result of changes in lifestyle and habits as well as the widespread use of screening technologies (2, 3). Unfortunately, many patients are detected at an advanced stage, which is especially typical in China, because PCa has a asymptomatic stage and lacks identifiable signs in its early stages (4, 5). Most cases of advanced PCa include bone metastases, particularly in the spine and pelvis (6). PCa that spreads to the bones not only causes skeletal-related events (SREs) like pathological fractures, bone pain, nerve and marrow compression, etc. but also severely lowers the quality of life and cancer survival rate by preventing patients from receiving radical surgery and limiting their treatment options to palliative and bone-protective measures (7, 8). Therefore, early detection of bone metastases from PCa is critical to improve patient outcomes. Currently, the most widely used technique for identifying bone metastases from PCa is the whole-body ^99m^Tc-MDP SPECT bone scan. Bone scanning is a sensitive but non-specific modality of bone scanning that can be easily influenced by benign bone alterations, such as infections, fractures, and benign bone degenerative lesions. As a result, false positive rates are rather high, and bone metastases cannot be reliably identified (9). Only 4% of men with newly diagnosed PCa had bone metastases, according to research from the United States (10). According to a Beijing study, bone metastases occurred in 21% of local individuals with newly diagnosed PCa (11). Currently, there are considerable variations in treatment approaches regarding whether patients with recently diagnosed PCa should have a bone scan right away because of regional variations in population epidemiology. Not only can unnecessary bone scans raise the risk of radiation exposure, but they also add to the expense of healthcare (11). Additionally, due to financial and technological limitations, bone scans and PET/CT examinations are not always possible in distant locations or primary hospitals. As a result, patients may not receive treatment for bone metastases in a timely manner and there is a chance that therapy may be delayed. Thus, a clinically grounded model for bone metastases in PCa needs to be created.

Numerous domestic and international research have demonstrated the tight relationship between the immune system, inflammatory response, and other variables and the development and progression of malignancies (12, 13). Inflammation-related oxidative damage may initiate cancer by leading to loss-of-function mutations in tumor suppressor genes or post-translational modifications of proteins involved in the control of DNA repair or apoptosis. Activation of inflammatory pathways may promote cell motility, vascular permeability, and angiogenesis to promote tumor development (14, 15). In addition, the inflammatory response in the tumor microenvironment contributes to the proliferation and survival of cancer cells and also plays an important role in tumor angiogenesis, metastasis, immune escape, and chemotherapy resistance (16). In recent years, scholars have discovered increasing inflammatory indicators that help diagnose and predict tumors, such as serum hypersensitivity C-react protein (hs-CRP), Fibrinogen (FIB), neutrophil-to-lymphocyte ratio (NLR), and platelet/lymphocyte ratio(PLR) (17–21). Since malignant tumors tend to cause poor nutrition and successive anemia, the 2004 European Cancer Anemia Survey also found that about 39% of cancer patients had anemia prior to treatment (22). Furthermore, low nutritional status and secondary anemia are frequently brought on by malignant tumors, and certain non-inflammatory markers like albumin and hemoglobin are also frequently utilized as prognostic indications for a bad prognosis for cancer (23–25). Systemic inflammation fuels PCa progression via IL-6/TNF-α pathways, elevating acute-phase proteins (e.g., CRP, fibrinogen) and suppressing nutritional markers (albumin, hemoglobin) (12, 14–16). We hypothesized that combining these biomarkers with clinical parameters (TPSA, Gleason) would enhance bone metastasis prediction.

The study established a predictive model for the total prostate-specific antigen (TPSA), high sensitive C-reactive protein-to-albumin ratio (HAR), Gleason grade groups (GG), hemoglobin (HB), and fibrinogen (FIB) as representatives of prostate carcinoma, and the model was internally validated to evaluate its predictability. The model was developed through screening of clinical pathological data, biochemical parameters, blood indicators, etc. in patients with local primary PCa.

## 2 Materials and methods

### 2.1 Study population

**Figure 1:**
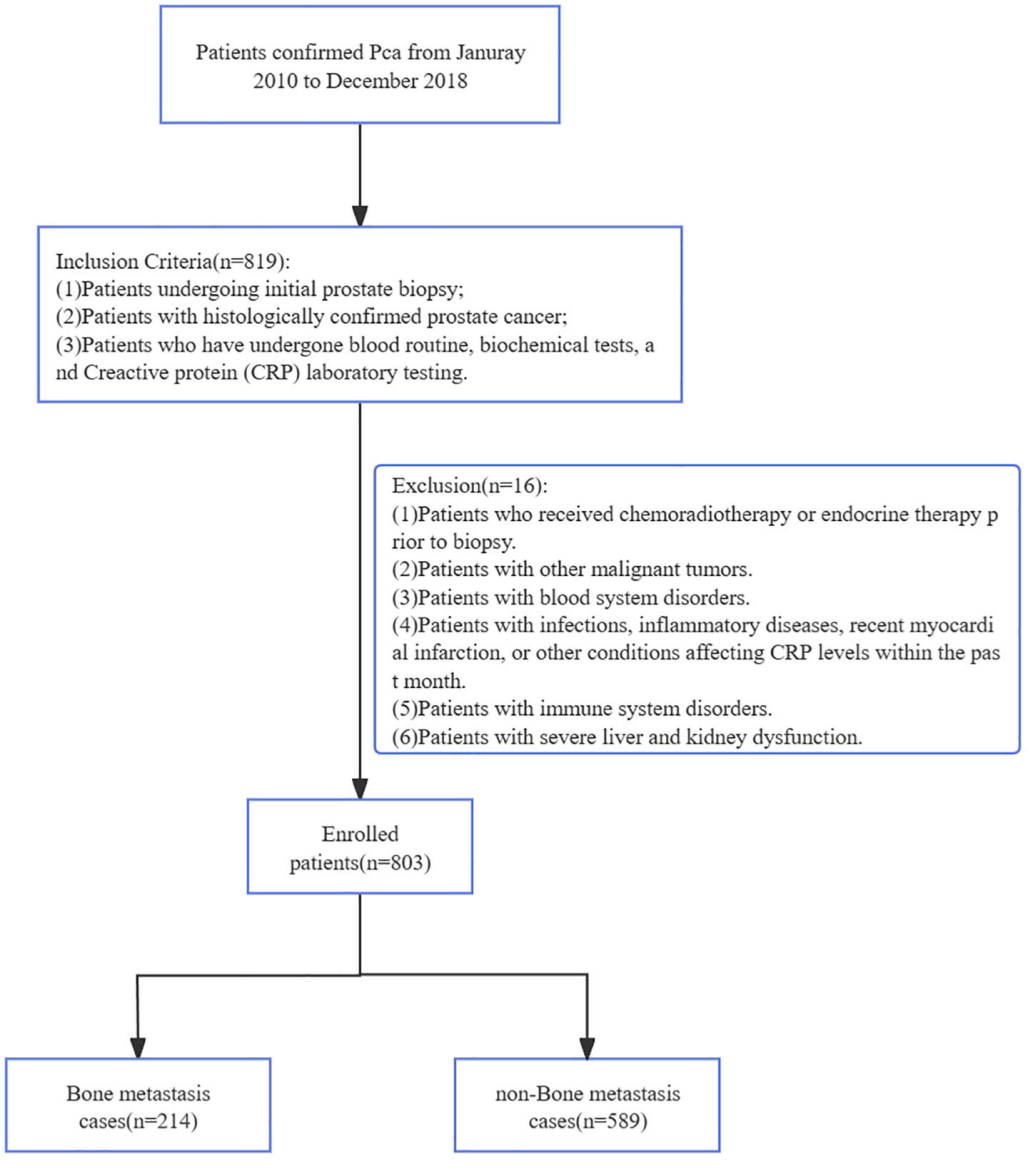
The simplified inclusion and exclusion criteria for PCa patient enrollment in the present study.

Clinical data were retrospectively extracted from the electronic medical record (EMR) system of The First Affiliated Hospital of Soochow University for all patients diagnosed with prostate cancer between January 2010 and December 2018. After initial identification of 1,202 potential cases, we applied sequential exclusion criteria to ensure data quality and study validity, ultimately enrolling 803 patients in the final analysis cohort (**Figure 1**). The EMR extraction included demographic information, laboratory test results, pathology reports, and imaging studies, with all personal identifiers removed prior to analysis to protect patient privacy. Inclusion criteria: (1)Patients undergoing initial prostate biopsy; (2)Patients with histologically confirmed PCa; (3)Patients who have undergone blood routine, biochemical tests, and CRP laboratory testing; Exclusion were applied sequentially: (1)Patients who received chemoradiotherapy or endocrine therapy prior to biopsy; (2)Patients with other malignant tumors; (3)Patients with blood system disorders; (4)Patients with infections, inflammatory diseases, recent myocardial infarction, or other conditions affecting CRP levels within the past month; (5)Patients with immune system disorders; (6)Patients with severe liver and kidney dysfunction (see **Figure 1**). This retrospective study was approved by the Institutional Review Board of The First Affiliated Hospital of Soochow University (Approval NO. 2024-073). Informed consent for study participation was waived (retrospective design). Consent for publication of identifiable data was obtained. Data are restricted due to privacy; requests require ethical approval.

There were 214 cases classified as having bone metastasis and 589 cases as not having bone metastasis based on whether bone metastases were present at the time of initial diagnosis. Between the two groups, there were no statistically significant differences in age, TG, or PLT, but there were statistically significant differences in hs-CRP, ALB, HAR, LMR, TPSA, PV, PSAD, HB, PLT, PLR, NLR, FIB, and Gleason grade groups (P < 0.05).

### 2.2 Diagnosis of prostate pathology and bone metastases

A transrectal or perineal prostate biopsy was performed on all 803 patients under the guidance of B-ultrasound. According to the International Society of Urological Pathology (ISUP) grading system in 2014, at least three pathologists with senior titles made all of the pathological diagnoses in the hospital. In ISUP groups 1-5, the Gleason scores are ≤6 points, 3 + 4=7 points, 4 + 3=7 points, 8 points, and 9~10 points, in that order. The quantity and location of bone metastases were ascertained using a ^99m^Tc-MDP full-body bone scan. MRI, PET/CT, CT, and additional techniques were utilized to confirm the diagnosis for patients whose suspicions of bone metastases could not be verified by a bone scan.

The procedures followed in this study were in accordance with the requirements of the World Medical Association Declaration of Helsinki revised in 2013.

### 2.3 Statistical methods

SPSS20.0 and R4.0.2 software were used for statistical analysis. The measurement data of normal distribution were represented by $\bar{x}$ ±s, and an independent sample T-test was used for component comparison. Measurement data with non-normal distribution are represented by M(Q1, Q3). Mann-Whitney U test was used. Categorical variables were expressed as frequencies and percentages (%), and comparisons between groups were performed using the Chi-square test or Fisher’s exact test, as appropriate. logistic regression was used to analyze the risk factors of bone metastasis. The prediction model of bone metastasis in newly diagnosed PCa was established by using R 4.0.2 software. the receiver operating characteristic (ROC) of each influencing factor and prediction model was plotted, and the AUC was calculated to evaluate the model’s discrimination ability. The AUC of different models was compared by the Delong test, and P < 0.05 was considered statistically significant. net reclassification improvement (NRI) and integrated discrimination improvement (IDI) were used to evaluate the ability of different models to improve classification efficiency. NRI > 0, IDI > 0 indicates positive improvement. The calibration curve and Hosmer-Lemeshow test were used to evaluate the accuracy of the model. decision curve analysis (DCA) was used to evaluate the clinical application value of the model, and the Bootstrap method was applied to repeat sampling 1000 times to verify the model internally. P < 0.05 was considered statistically significant.

### 2.4 Development of prediction models

**Figure 2:**
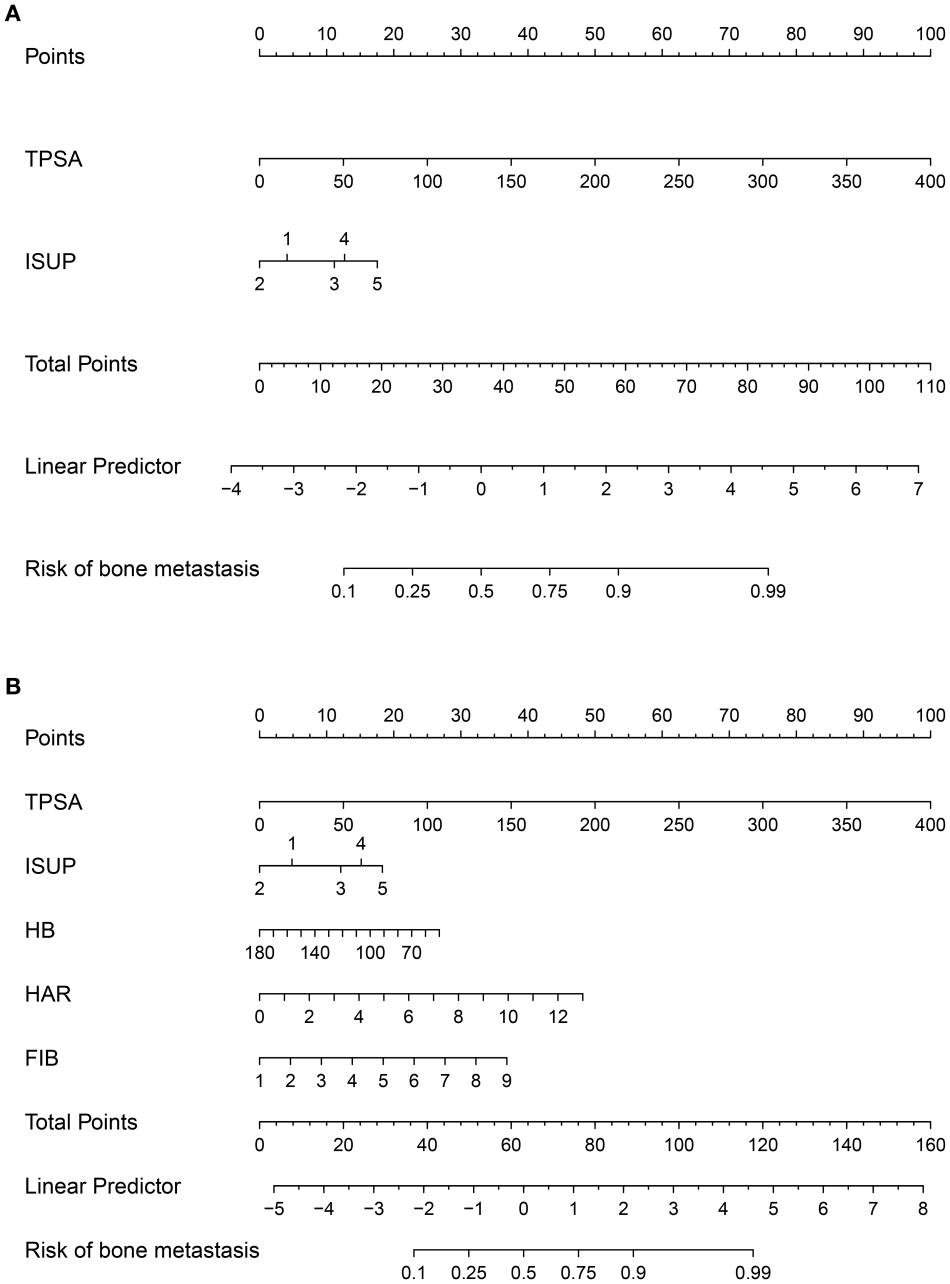
Nomograms for predicting bone metastasis in newly diagnosed prostate cancer patients. **(A)** Clinical model based on TPSA and ISUP grade groups. **(B)** Comprehensive model incorporating inflammatory markers (HAR, FIB) and hemoglobin (HB) in addition to clinical parameters.

Based on the independent predictors identified by the multivariate logistic regression analysis, we constructed two distinct prediction models: a Clinical Model (incorporating TPSA and ISUP grade groups) and a Comprehensive Model (additionally incorporating HAR, FIB, and HB). The rms package in R was used to generate the corresponding nomograms (**Figure 2**), which translate the regression coefficients into a user-friendly points-based system for estimating the probability of bone metastasis.

## 3 Results

### 3.1 Demographic and laboratory indicators

A total of 803 patients newly diagnosed in our hospital with pathologically confirmed PCa were included in this study, with a median age of 71 years, and the median HAR of 589 patients with non-bone metastatic PCa was 0.029 × 10^-4^ (0.014 ~ 0.067 × 10^-4)^. The median HAR of 214 patients with bone metastasis PCa was 0.159 × 10^-4^ (0.0527 ~ 0.343 × 10^-4^), and the HAR value of patients with bone metastasis PCa was higher than that of patients without bone metastasis PCa, and the difference was statistically significant (P < 0.01). The bone metastasis group showed statistically significant differences in TPSA, FIB, HAR, HB, and Gleason grade groups (P < 0.05), while there were no statistically significant differences in other indicators (see **Table 1**). According to the 2014 ISUP grading system, the bone metastasis rates were 5.4% (6/112) in Group 1, 7.0% (10/143) in Group 2, 22.3% (31/139) in Group 3, 28.2% (42/149) in Group 4, and 46.1% (125/271) in Group 5.

**Table 1:** The comparison of clinical data between patients with bone metastatic PCa and patients with non-bone metastatic PCa.

Variables	Non-bone metastatic PCa (n=589)	Bone metastatic PCa (n=214)	Stats	P -value
Age (years)	70.66±7.99	71.94±7.41	t=2.056	0.04
hs-CRP (mg/l)	1.14 (0.57∼2.71)	6.03 (2.22∼13.41)	z=-11.529	<0.001
ALB (g/l)	42.40 (39.50∼45.00)	39.05 (36.40∼42.40)	z=-8.665	<0.001
HAR (10^-4^)	0.029 (0.014∼0.067)	0.159 (0.527∼0.343)	z=-11.715	<0.001
LMR	3.88 (2.90∼5.09)	3.45 (2.52∼4.50)	z=-3.520	<0.001
TPSA (ng/ml)	19.40 (10.77∼43.44)	100.00 (44.36∼100.00)	z=-13.458	<0.001
TPV (ml)	35.64 (24.92∼51.47)	37.67 (25.97∼54.36)	z=-3.542	<0.001
PSAD (ng/ml^2^)	0.69 (0.33∼1.36)	0.88 (0.39∼1.87)	z=-9.129	<0.001
HB (g/l)	142 (131∼151)	129 (112∼142)	z=-9.179	<0.001
WBC (10^9^/l)	5.66 (4.90∼6.75)	6.13 (4.90∼7.45)	z=-2.205	0.027
PLT (10^9^/l)	185 (151∼221)	189 (144∼251)	z=-1.572	0.116
NLR	2.29 (1.68∼3.06)	2.63 (1.91∼3.65)	z=-3.573	<0.001
PLR	119.52 (91.32∼158.50)	130.06 (99.02∼179.41)	z=-2.838	0.005
FIB (g/l)	2.66 (2.27∼3.17)	3.40 (2.70∼4.50)	z=-9.796	<0.001
TG (mmol/l)	1.13 (0.81∼1.64)	1.12 (0.86∼1.45)	z=-0.454	0.650

hs-CRP, highly sensitive C-reactive protein; ALB, albumin; HAR, the ratio of highly sensitive C-reactive protein/albumin; LMR, the ratio of hemlymphocyte to monocyte; tPSA, total prostate-specfic antigen; TPV, prostate volume; PSAD, PSA density; HB, hemoglobin; WBC, blood leukocyte; PLT, the platelet count; NLR, the ratio of neutrophils to lymphocytes; PLR, the ratio of platelets to lymphocytes; FIB, fibrinogen; TG, triglyceride.

### 3.2 Univariate and multivariate logistic regression analysis of bone metastases in newly diagnosed PCa patients

Taking the occurrence of bone metastasis in patients with newly diagnosed PCa as the dependent variable, the above statistically significant indicators were included in univariate and multivariate logistic regression analysis, The results of the univariate analysis showed that the hs-CRP (OR=1.190, P < 0.001), ALB (OR=0.857, P < 0.001), HAR (OR=1.926, P < 0.001), LMR (OR=0.842, P < 0.001), ALB (OR=0.857, P < 0.001), HAR (OR=1.926, P < 0.001), LMR (OR=0.842, P < 0.001), TPSA (OR=1.029, P < 0.001), PV (OR=1.011, P < 0.001), PSAD (OR=1.951, P < 0.001), HB (OR=0.958, P < 0.001), PLT (OR=1.003, P=0.005**),** PLR (OR=1.004, P=0.001), NLR (OR=1.113, P=0.003), FIB (OR=2.305, P < 0.001), Gleason grade groups were statistically significant. The results of stepwise forward regression demonstrated that individuals with recently diagnosed PCa, TPSA, FIB, HB, HAR, and Gleason grades were independent influencing factors for bone metastasis (P < 0.05; **Table 2**). Odds Ratios for ISUP groups are relative to ISUP Group 1 (reference group). The decreasing OR from Group 2 to Group 5, combined with the increasing actual metastasis rate (5.4% to 46.1%), indicates a complex risk relationship best interpreted by the nomogram. There is no collinearity between the variables, as indicated by the variance expansion factors, which are all smaller than 5. Consequently, the independent influencing factors mentioned above are included in the model’s formulation.

**Table 2:** Multivariate logistic regression analysis of predictors for bone metastasis in newly diagnosed PCa.

Variables	Regression	OR (95%CI)	Wald	P -value
HAR (10^-4^)	0.311	1.365 (1.146-1.626)	12.158	<0.001
TPSA (ng/ml)	0.021	1.021 (1.016-1.027)	61.317	<0.001
HB (g/l)	-0.17	0.983 (0.972-0.995)	8.412	0.004
FIB (g/l)	0.388	1.473 (1.156-1.878)	9.815	0.002
ISUP 1 (reference)			18.899	0.001
ISUP 2	-1.131	0.323 (0.125-0.833)	5.693	0.019
ISUP 3	-1.537	0.215 (0.100-0.463)	15.390	<0.001
ISUP 4	-0.521	0.594 (0.335-1.053)	3.178	0.075
ISUP 5	-0.265	0.767 (0.456-1.289)	1.004	0.316

HAR, the ratio of highly sensitive C-reactive protein/albumin; HB, hemoglobin; FIB, fibrinogen; ISUP, International Society of Urological Pathology; Odds Ratios for ISUP groups are relative to ISUP Group 1 (reference group). The decreasing OR from Group 2 to Group 5, combined with the increasing actual metastasis rate (5.4% to 46.1%), indicates a complex risk relationship best interpreted by the nomogram.

### 3.3 The establishment of a prediction model and the efficacy of diagnosis and treatment

**Figure 3:**
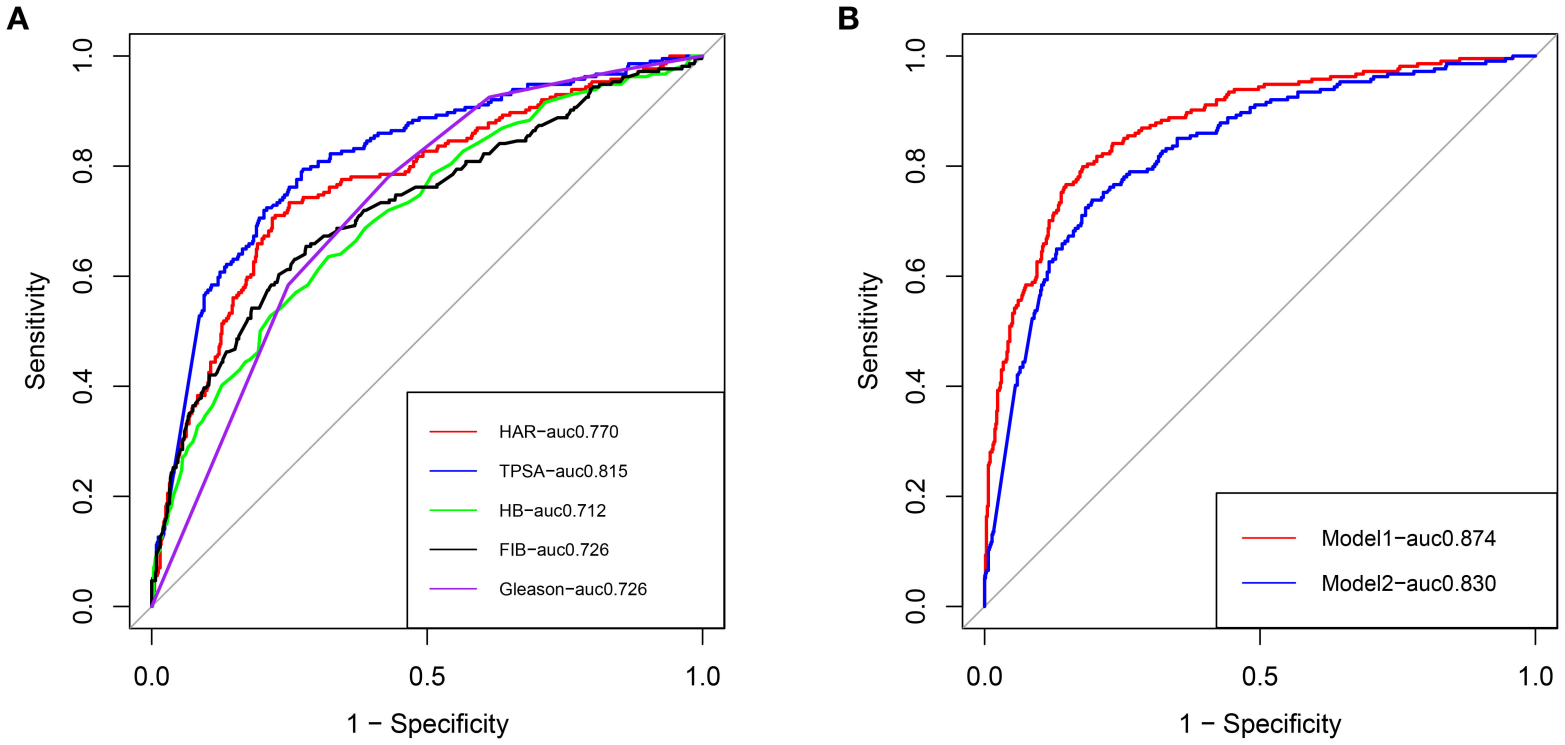
**(A)** ROC curves of clinical risk factors for predicting bone metastasis in patients with newly diagnosed PCa. **(B)** ROC curves of the clinical prediction model and comprehensive prediction model for predicting bone metastasis in patients with newly diagnosed PCa, Red: Comprehensive model; Blue: Clinical model.

**Figure 4:**
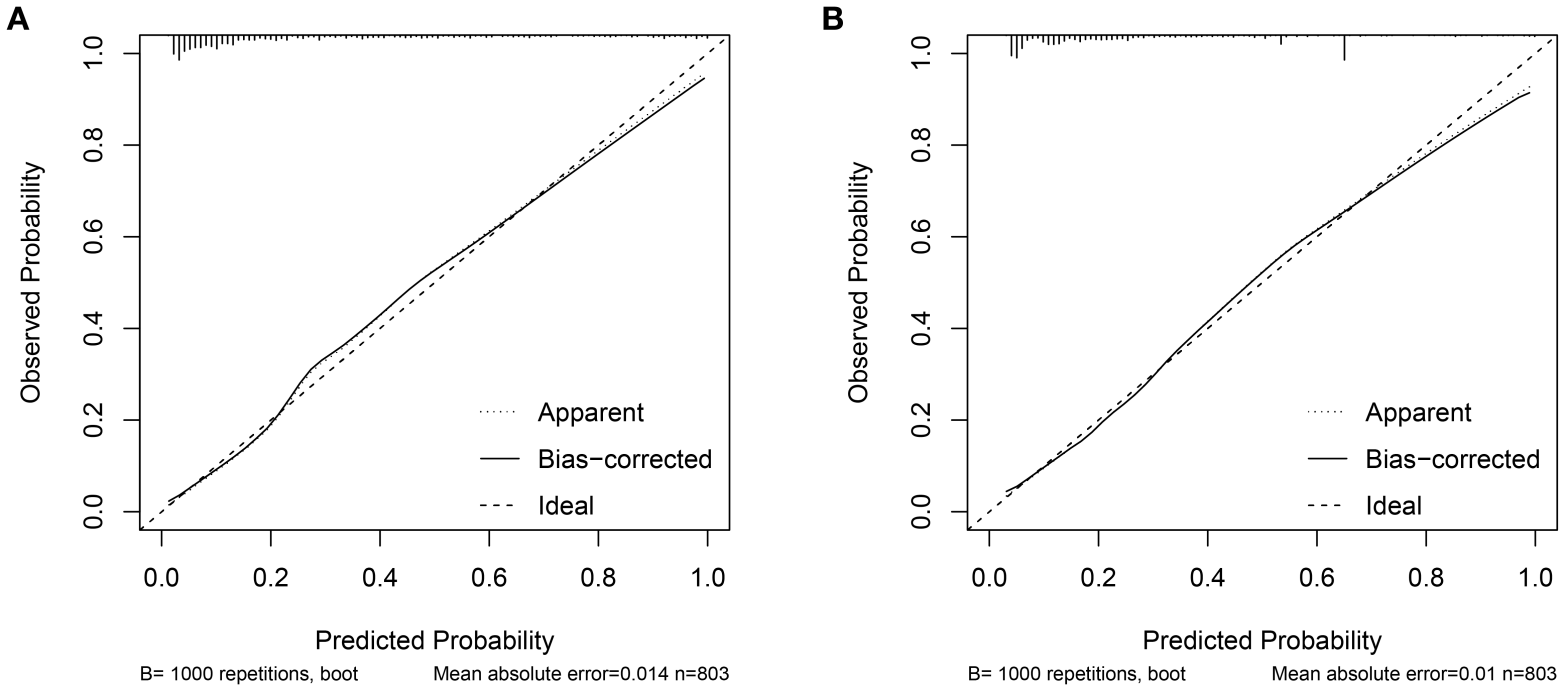
**(A)** Comprehensive model calibration curve. **(B)** Clinical model calibration curve.

Two nomograms were created: a clinical nomogram based on clinicopathological data and a comprehensive nomogram with parameters like inflammatory indicators (see **Figure 2**). The five statistically significant components in the multivariate logistic regression analysis above served as parameters. All influencing factors and models for predicting bone metastases in patients with newly diagnosed PCa were plotted on a ROC (**Figure 3**, **Table 3**). TPSA was found to have the greatest predictive performance among the contributing factors mentioned above, with an AUC of 0.814 (95% CI 0.780-0.849), 79.4% sensitivity, and 72.5% specificity. The inflammatory indicator with the strongest predictive performance that we looked at was HAR. The sensitivity was 70.6%, the specificity was 78.1%, and the AUC was 0.770 (95% CI 0.732-0.808). The clinical model’s AUC was 0.830 (95% CI 0.798-0.863), while the comprehensive model’s AUC was 0.874 (95% CI 0.845-0.902), suggesting that both models possessed strong discriminating abilities. The comprehensive model’s prediction findings were found to be highly consistent with the actual clinical observation data, as demonstrated by the Hosmer-Lemeshow test (x^2^=9.312, P=0.317). The clinical model (P=0.995, x^2^=1.333). The calibration plot demonstrated that there was strong agreement between the two models’ projected and actual observed results (**Figure 4**).

**Table 3:** The diagnostic efficacy of HAR, PSA, HB, FIB, Gleason grading group, and model for bone metastasis of PCa were compared.

Variables	AUC(95%CI)	Youden index	Cut-off value	Sensitivity	Specificity
HAR(10^-4^)	0.770 (0.732,0.808)	0.49	0.75	70.6%	78.1%
TPSA(ng/mL)	0.814 (0.780,0.849)	0.52	37.77	79.4%	72.5%
HB(g/l)	0.712 (0.671,0.753)	0.31	134.5	63.6%	67.8%
FIB(g/l)	0.726 (0.684,0.768)	0.374	3.08	65.4%	72.0%
ISUP	0.726 (0.691,0.762)	0.35	3.5	78.0%	57.1%
Comprehensive model	0.874(0.845,0.902)	0.62	–	79.9%	82.2%
Clinical model	0.830(0.798,0.863)	0.27	–	73.8%	80.5%

HAR, the ratio of highly sensitive C-reactive protein/albumin; HB, hemoglobin; FIB, fibrinogen.

### 3.4 Clinical application and internal validation of the prediction model

**Figure 5:**
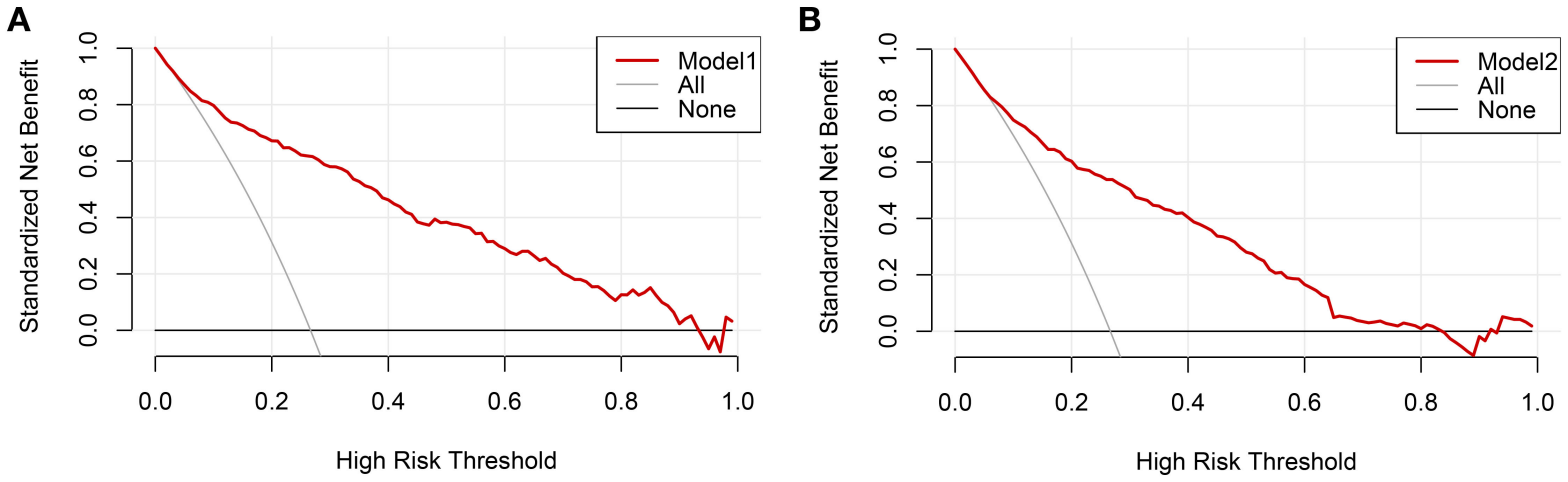
**(A)** Comprehensive model decision curve **(B)** Clinical model decision curve.

**Figure 6:**
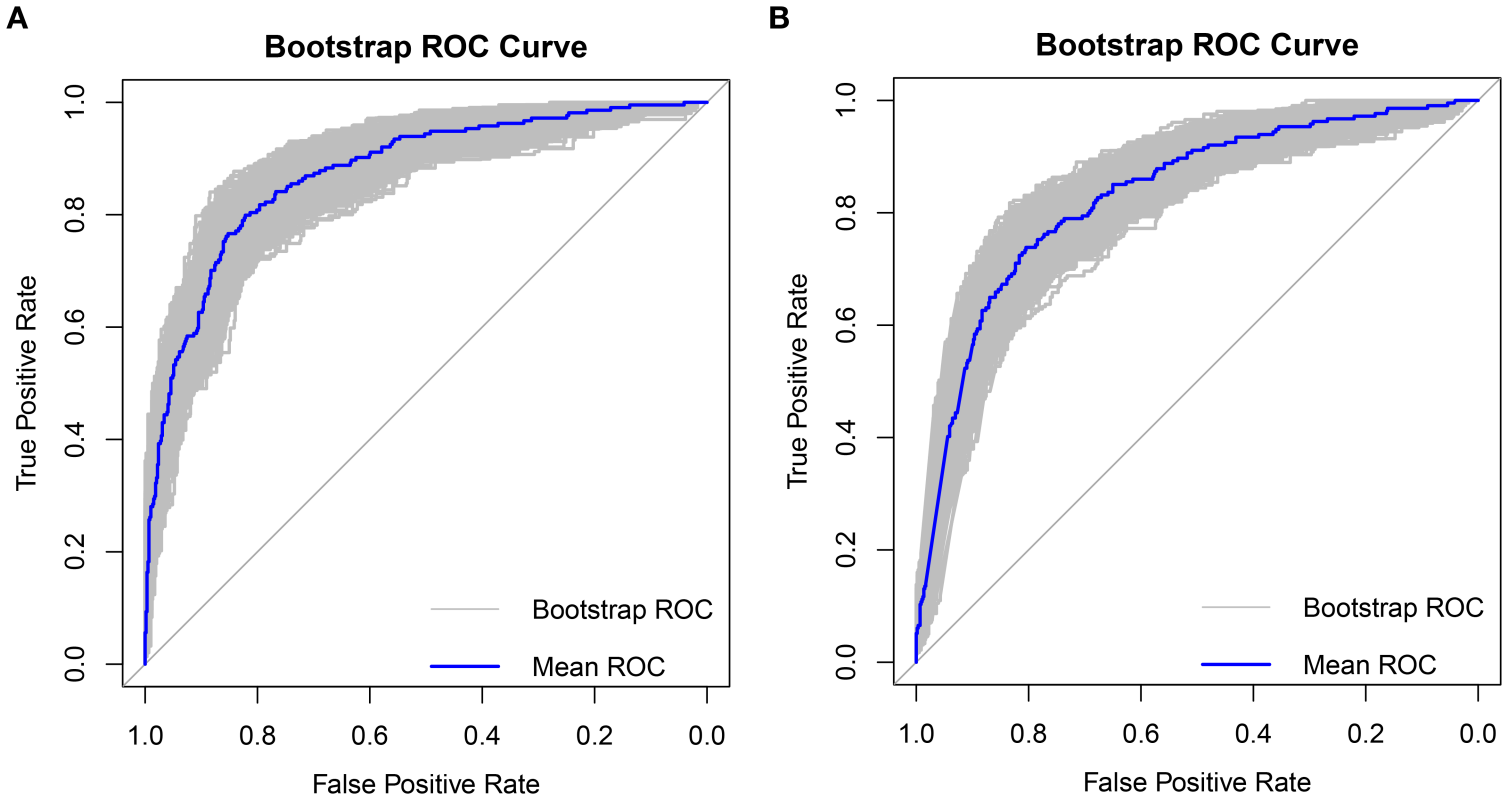
Bootstrap internal validation 1000 times ROC plot **(A)** Comprehensive model mean AUC was 0.868, **(B)** Clinical model mean AUC was 0.826.

The clinical decision curves for each of the prediction models were created in order to assess the two models’ actual clinical application effects (**Figure 5**). The comprehensive model’s DCA curve was greater than the two extreme values while its prediction probability ranged from 5% to 90%, which provided patients with additional therapeutic benefits. The DCA curve was greater than the two extremes when the clinical model’s prediction probability ranged from 10% to 80%, which was more advantageous for the patients. The ROC curves for the two models were constructed for 1000 iterations of Bootstrap following internal verification using Bootstrap (**Figure 6**). The clinical model’s mean AUC value was 0.826 and the comprehensive model’s mean AUC value was 0.868, demonstrating the strong consistency between the two models. The comprehensive model outperformed the clinical model in terms of predictive capacity, as evidenced by the statistically significant (P < 0.01) difference between the two models according to the findings of the Delong test. The comprehensive model was shown to be much better than the clinical model, as evidenced by its 8.96% improvement in reclassification accuracy and 74.46% continuous NRI when compared to the clinical model. With an IDI of 0.103 (95% CI 0.075-0.130, P < 0.01), the integrated model outperformed the clinical model in terms of prediction accuracy by 10.3%.

## 4 Discussion

In our cohort of 803 PCa patients, 26.7% (214/803) had bone metastasis at diagnosis, which is comparable to reports from Beijing (21%) but higher than United States data (4%) (10). For Chinese patients with PCa, early diagnosis of bone metastases is very crucial. According to studies, bone metastases, the primary cause of morbidity and mortality in PCa patients, account for 90% of the deaths of PCa patients (26). Patients with PSA > 20ng/ml, poorly differentiated, and asymptomatic PCa are advised to have bone scans, according to the European Society of Urology (27). In the Chinese expert consensus on clinical diagnosis and treatment of bone metastases and bone-related diseases of PCa, When bone-related symptoms including pain, fractures, symptoms connected to the spinal cord, or nerve compression occur, blood alkaline phosphatase levels are raised. Hypercalcemia: For patients with newly diagnosed PCa who have a Gleason score of ≥8 or a clinical stage of ≥T3, a bone scan is advised (8). The lack of a global standard for bone scan evaluation in patients with early PCa exists at the moment, and this is also linked to regional variations in population epidemiology.

The human body initiates an inflammatory response in reaction to injury or infection, which triggers the production of IL-1, IL-6, IL-8, TNF-α, and other inflammatory factors by white blood cells. These factors aid in the body’s tissue repair process. Among the most well-known inflammatory cytokines is IL-6. One of IL-6’s primary actions is to promote the liver’s synthesis of CRP and other acute-phase proteins, which are then released into the bloodstream. These proteins include ferritin, fibrinogen, and serum amyloid A and P components. As was already mentioned, there is a correlation between increased inflammatory factor production and the development and progression of tumors. Tumor metastasis is significantly influenced by the epithelial-mesenchymal transition (EMT), Regarding the mechanism underlying PCa’s bone metastasis, one idea holds that IL-6 controls EMT and the cancer cells’ homing to the bone (28). Prior research has demonstrated a strong correlation between increased levels of TNF-α and IL-6 and metastatic PCa (29). Kim et al. found that the interaction between tumor cell IL-6 and macrophage TNF-α promoted the growth of human PCa cells within the bone of nude mice (30). Chen et al. found that the YY1 complex in M2 macrophages promotes PCa progression through up-regulation of IL-6 (31). Thus, tumor and inflammation are closely related, and new evidence for the formation or advancement of PCa has been produced by epidemiological, histological, and molecular pathological studies (32–34).

We can therefore conclude that the body’s inflammatory factors will be slightly elevated during the early stages of a tumor. Although CRP is a more extensively used and frequently used inflammatory marker in clinical practice, it is typically quite difficult to detect and very low in healthy individuals. The slightly elevated increase in CRP is difficult to identify with regular laboratory detection methods. Currently, the body’s extremely low concentration of CRP may be precisely detected using laboratory hypersensitive detection technology. This sensitive measure is used to assess the low-level inflammatory condition; it is known as high-sensitivity CRP (hs-CRP) (14). according to our previous study, Patients with elevated hs-CRP had a higher rate of positive prostate biopsies (35), It is an independent risk factor for bone metastasis in patients with PCa (36). According to Zhou et al., PCa patients had lower overall survival (OS), cancer-specific survival (CSS), and progression-free survival (PFS) when their CRP levels were greater (37). The primary synthesis of fibrinogen (FIB), a crucial marker protein in the human body’s coagulation process, occurs in the liver. Malignant tumors and inflammation raise the level of FIB, which builds up at the tumor site to generate high expression and influence tumor growth. Additionally, FIB is thought to be a poor prognostic factor for many malignant cancers, including malignancies of the urinary system, gynecological system, and digestive system (38–40). For the first time in the history of PCa, we discovered in a prior study that the prior to treatment level of FIB was correlated with the number of bone metastases and constituted a separate risk factor for a high burden of bone metastases in PCa. In line with our findings (41), Yu et al. discovered that FIB had extra predictive value for bone metastases in PCa (21). The interaction of fibrin, platelets, and tumor cells to form platelet-fibrin-tumor cell aggregates, promote vascular endothelial cell adhesion and metastatic spread, and promote tumor cell growth and survival is one current theory regarding how FIB contributes to the progression and metastatic potential of PCa (42). When malignant tumors grow to an advanced stage or spread, they frequently result in secondary anemia and cancer-related cachexia, Anemia and hemoglobin levels were found to be strongly correlated with the survival rate of cancer patients, according to a meta-analysis of 60 clinical studies. The risk of death was higher for cancer patients with anemia (65%) and PCa (47%) (43). A study comprising 91 cervical cancer cases discovered a correlation between low hemoglobin and elevated levels of reactive oxygen species, CRP, IL-1, TNF-α, ß, and IL-6, multivariate analysis revealed that IL-6 was a separate factor influencing hemoglobin levels (44). By decreasing HB’s ability to absorb iron through the upregulation of hepcidin, IL-6 may exacerbate anemia (45). An essential marker of liver function and nutritional condition is albumin (ALB). Low levels of albumin are frequently observed in cancer patients, which may indicate a lower nutritional condition and may disrupt immunological processes like phagocytosis and humoral and cellular immunity (46). An essential marker of liver function and nutritional condition is albumin (ALB). Low levels of albumin are frequently observed in cancer patients, which may indicate a lower nutritional condition and could affect immunological processes like phagocytosis and humoral and cellular immunity. Additionally, during the early stages of tumor formation, albumin starts to decline. Two possibilities are offered. One explanation is that inflammatory substances like IL-6 either stimulate vascular permeability and albumin loss in blood vessels into the tissue fluid (47), or they may prevent albumin from being produced (48). Consequently, we discovered that bone metastasis resulting from PCa is an intricate biological process triggered by multiple causes. The interaction of chemokines, macrophages, and other growth factors is crucial to the malignant evolution of PCa.

The C-reactive protein-to-albumin ratio (CAR) has gained growing attention in recent years as a novel indicator of the systemic inflammatory response. It is valuable in assessing the inflammatory status of individuals with different disorders than just CRP and ALB. Research has demonstrated a correlation between CAR and the prognosis of multiple malignancies, including gastric, endometrial, gallbladder, and pancreatic tumors (49–53). There are, however, not many investigations on cancers of the urinary system, particularly PCa. The preceding study by Taizo et al. showed for the first time that CAR was an independent predictor of OS and CSS in patients with castration-resistant prostate cancer (CRPC) and that individuals with high CAR had shorter CSS for patients with newly diagnosed castration-resistant PCa taking abiraterone or enzalutamide (54). For the first time, we looked into the connection between HAR and bone metastases using HAR in our investigation.

In this analysis, we included 15 possible predictors, which was a considerable increase over the number of predictors included in previous studies. These included inflammatory markers and clinicopathological data. HAR, FIB, HB, PSA, and Gleason grade groups were found to be independent predictors of bone metastases in newly diagnosed PCa, according to univariate and multivariate logistic regression analysis. Based on clinicopathological data (PSA, Gleason grade group), we constructed a clinical prediction nomogram and a comprehensive prediction nomogram that included HAR, FIB, and HB. The clinical nomogram’s AUC was 0.830, whereas the complete nomogram’s AUC was 0.874. The Delong test revealed a significant difference in AUC values between the two models (P < 0.01). Moreover, the comprehensive model’s performance was assessed using IDI and NRI. The predictive performance of the comprehensive model was 10.3% better than the clinical model, and its reclassification accuracy was 8.96% higher than the clinical model’s. The mean AUC of the comprehensive model, as determined by Bootstrap internal verification, was 0.868, demonstrating the model’s strong consistency. Our HAR AUC (0.770) aligns with Zhou et al. (37), while Yu et al. similarly validated fibrinogen’s predictive role. The comprehensive model’s AUC (0.874) surpasses clinical-only tools like Briganti’s nomogram (AUC 0.82) (27). The potential of the previously indicated mechanism of PCa bone metastases was also supported by our conclusion. PCa metastasis is directly linked to elevated IL-6 and TNF-α levels, which in turn drive an upsurge in CRP and FIB levels in the body, prevent iron from being absorbed as the building block of hemoglobin, and worsen anemia. Vascular permeability increases due to the body’s long-term, modest rise in inflammatory factors. As a result, materials like albumin are lost from blood vessels into the interstitial fluid, aggravating the body’s malnutrition state and promoting the development of tumors. When making an initial diagnosis, it is simple to obtain these laboratory tests right away. It is simple to advertise in outlying locations and main hospitals. Using this approach, doctors may immediately determine a patient’s risk of bone metastases if they have recently been diagnosed with PCa. Consequently, in order to maximize benefits, it can offer patients with probable bone metastases of PCa individualized care and therapy.

Our study has several limitations that should be considered. First, as a single-center retrospective analysis lacking external validation, the potential for selection bias cannot be excluded. Second, although we adjusted for age and body mass index, data on certain comorbidities (e.g., cardiovascular disease and diabetes) and lifestyle factors (such as smoking history) were not uniformly available due to the retrospective nature of the study. Therefore, residual confounding from these unmeasured variables may remain. Third, important clinical variables including prostate MRI findings and serum alkaline phosphatase levels were not incorporated into the model, as these were not routinely performed for all patients during the study period (2010–2018), and their inclusion could have introduced selection bias. Finally, the lack of long-term follow-up data precluded analysis of the associations between inflammatory markers and survival or disease progression outcomes. Thus, large-scale, multi-center prospective studies are warranted to validate our nomogram and further elucidate the prognostic value of inflammatory markers such as HAR, FIB, and HB in prostate cancer.

In conclusion, non-invasive, practical, and affordable laboratory tests, such as serum PSA and other blood indicators, are standard procedures for patients with PCa who are admitted to hospitals. The study’s findings demonstrated that among patients with newly diagnosed PCa, HAR, FIB, HB, TPSA, and Gleason score grade group at biopsy were independent influencing variables for bone metastasis. In individuals with recently diagnosed PCa there is a substantial probability of bone metastases when TPSA >37.7ng/ml, HAR >0.75×10-4, FIB >3.08g/L, HB <134.5g/L, and Gleason score ≥8 are present. In comparison to a single clinical model, we think the comprehensive model created by integrating inflammatory markers and other factors with TPSA and Gleason grade groups has a higher predictive efficiency and can increase both diagnostic and predictive efficiency.

## Data Availability

The datasets presented in this article are not readily available because participants refuse. Requests to access the datasets should be directed to 962876336@qq.com.
